# Perceived social support, academic buoyancy, and L2 willingness to communicate among Chinese EFL undergraduates: a structural equation modeling study

**DOI:** 10.3389/fpsyg.2026.1920700

**Published:** 2026-10-19

**Authors:** Xiao Zhu, Nur-Ehsan Mohd-Said, Chairozila Bt. Mohd. Shamsudin

**Affiliations:** 1 Faculty of Education, Universiti Kebangsaan Malaysia (UKM), Bangi, Malaysia; 2 School of Foreign Languages, Shangrao Normal University, Shangrao, China; 3 Pusat Pengajian Citra Universiti, Universiti Kebangsaan Malaysia (UKM), Bangi, Malaysia

**Keywords:** academic buoyancy, EFL undergraduates, perceived social support, structural equation modeling, willingness to communicate

## Abstract

**Introduction:**

Willingness to communicate (WTC) stands as a critical factor influencing learners’ practical use of a second language (L2). To date, few studies have systematically analyzed the psychological pathways between contextual social support and L2 WTC, with insufficient attention paid to learners’ adaptive capabilities. This study explored how perceived social support from teachers, parents, and peers correlates with learners’ L2 communicative willingness and examined the mediating function of academic buoyancy in this relationship.

**Methods:**

Using a sample of 369 first-year non-English-major undergraduates in eastern China, structural equation modeling (SEM) alongside 5,000 bootstrap resamples was conducted to analyze the structural relationships among social support sources, academic buoyancy, and L2 WTC.

**Results:**

Results indicated that all three forms of social support were positively associated with both academic buoyancy and WTC. Among these, teacher support showed the strongest standardized association with WTC, followed by peer and parent support. Academic buoyancy was found to partially mediate the associations between each source of social support and WTC, with teacher support showing the largest indirect effect (15.32%).

**Discussion:**

These findings contribute to WTC literature by positioning academic buoyancy as an important adaptive psychological factor that links contextual support to communicative readiness. The results further highlight the differentiated functions of teacher, peer, and parent support within Chinese EFL contexts. Corresponding pedagogical implications are proposed to establish supportive, resilience-oriented language learning environments.

## 1 Introduction

Willingness to communicate (WTC) has been widely regarded as a pivotal construct for understanding why some language learners actively participate in second language (L2) interaction while others remain reluctant, even with comparable linguistic competence (MacIntyre and Legatto, 2011; Peng, 2025). Originating as a relatively stable dispositional trait in first language (L1) communication research (Bensalem et al., 2025; MacIntyre et al., 2019a; Yang and Lian, 2023), WTC was later reconceptualized for L2 acquisition as a situation-responsive readiness to initiate communication with specific interlocutors at a given moment (MacIntyre et al., 1998). As the immediate precursor to actual L2 use, WTC serves as a psychological bridge that enables learners to transform their linguistic knowledge into communicative action (Lee, 2022).

Building on this conceptualization, recent research has increasingly shifted from viewing WTC as a fixed dispositional trait to viewing it as a non-static concept susceptible to both real-time contextual variables and social-cultural interactions (Dewaele, 2019; Shen et al., 2024; Zhang et al., 2024). In EFL classrooms, learners’ WTC emerges from the interplay between personal dispositions and external contextual factors. Relevant studies have shown that perceived communicative competence and foreign language anxiety, two key internal variables, exert notable impacts on learners’ WTC (Lin et al., 2025; MacIntyre et al., 2002; MacIntyre and Gregersen, 2022), alongside various external situational factors (Han and Li, 2025; Wang et al., 2021). Scholars have gradually come to recognize that contextual factors play a tangible role in facilitating or limiting L2 interaction among learners.

Perceived social support is one critical contextual factor that strongly correlates with learners’ emotional states, classroom participation and actual communication performance (Collie et al., 2017; MacIntyre et al., 2001; Wang et al., 2023). Specifically, the variable reflects how students subjectively evaluate the affective, cognitive, and instrumental assistance obtained from instructors, classmates, and family members (Zimet et al., 1988). When learners feel adequately supported, they tend to experience less language learning anxiety, participate more actively, and become more willing to engage in interactive communication (Liu and Hong, 2021; Zarrinabadi and Khodarahmi, 2024). Prior empirical work further indicates that sound interpersonal connections within classrooms can boost learners’ WTC, especially during meaning-focused activities (Li Z. et al., 2025).

Accumulated research evidence has validated the connection between perceived social support and L2 learners’ WTC. Even so, few investigations have clarified the psychological pathways that explain how social support helps learners keep a stable state of communicative willingness. Most existing literature prioritizes affective variables, such as enjoyment and anxiety as mediating variables (Acosta-Manzano et al., 2024; Dewaele et al., 2023; Wang and Hui, 2024; Xiao and Jia, 2025). Recent work has further broadened this line of inquiry by examining how regret about past experiences relates to EFL learners’ motivation and WTC through positive and negative emotions (Almubarak and Alikhani, 2026). Such findings reinforce the relevance of examining psychological mechanisms alongside contextual factors when explaining variation in L2 WTC. Nevertheless, comparatively less attention has been paid to learners’ ability to cope with routine academic difficulties and frustrations (Souzandehfar and Ahmed Abdel-Al Ibrahim, 2023; Yun et al., 2018; Zhai, 2025). In this regard, academic buoyancy offers a viable theoretical framework to address this gap.

Academic buoyancy characterizes learners’ capacity to contend with recurring academic stress (Martin and Marsh, 2008), which differs from resilience, a trait associated primarily with responses to severe adversities. Individuals with higher academic buoyancy can better sustain learning motivation, regulate negative emotions, and keep active participation in classroom activities (Xu and Wang, 2022; Yang and Liang, 2025). Although prior work has validated academic buoyancy as a mediator connecting environmental resources to learning outcomes (Datu and Yang, 2021; Putwain and Wood, 2023), its role in L2 learners’ WTC has not been thoroughly explored (Yun et al., 2018). This research gap is especially obvious in China’s EFL education settings. Local sociocultural features, including hierarchical classroom culture, face concerns and high parental expectations, all interfere with learners’ choices in language communication (Cortazzi and Jin, 1996; Cui and Mahfoodh, 2026; Wen and Clément, 2003). Within such contexts, students’ WTC may rely not merely on their affective states but also on their ability to adapt to daily academic and sociocultural requirements.

Against this background, the present study proposed and tested a structural equation model. This model examined the direct and indirect effects of social support from teachers, parents, and peers on WTC, with academic buoyancy acting as the mediating variable among Chinese tertiary EFL learners. By integrating these constructs within a socially embedded language learning framework, this study delivers three advancements to existing WTC research. First, it extends current theoretical frameworks of WTC by incorporating academic buoyancy, an adaptive capacity construct that has received limited attention in L2 communication research. Second, it identifies adaptive coping capacity as an important psychological pathway linking contextual support to communicative willingness, moving beyond the dominant focus on affective mediators such as anxiety and enjoyment. Third, it differentiates the distinct roles of teacher, parent, and peer support, delivering deeper insight into how multisource social support is associated with learners’ WTC.

## 2 Literature review

### 2.1 Willingness to communicate

Initial L1 communication models treated WTC as a static predisposition, characteristically driving a person’s enduring readiness or reluctance to start conversations (Burgoon, 1976; McCroskey and Baer, 1985). In the field of SLA, MacIntyre et al. (1998) reconceptualized WTC as learners’ situational preparedness to initiate a conversation with a specific individual or group using an L2. This definition marked an important theoretical shift by emphasizing the situational and contextual dimensions of communicative willingness. Subsequent studies have increasingly conceptualized WTC as a dynamic construct responsive to interpersonal relationships, affective states, and situational conditions (MacIntyre et al., 2019b; Yang and Lian, 2023). Often described as a “psychological gatekeeper” to actual language use (Lee, 2022), WTC represents the final volitional stage preceding communication and is closely associated with learners’ opportunities for L2 interaction and their overall learning outcomes (Dewaele, 2019; Zhang et al., 2024).

Empirical studies have identified numerous antecedents of WTC. Research has gradually moved from an early emphasis on internal variables, such as self-esteem and perceived communicative competence (Leeming et al., 2024; MacIntyre, 1994; Zhou et al., 2023), toward more ecologically oriented perspectives that recognize the interaction between individual characteristics and contextual conditions (Peng, 2012; Wang et al., 2021). Within this ecological perspective, two conceptually complementary yet seldom integrated constructs warrant particular attention: perceived social support, which reflects learners’ external contextual resources, and academic buoyancy, which represents learners’ internal adaptive capacity to translate supportive environments into sustained communicative engagement.

### 2.2 WTC and perceived social support

Extensive research on WTC has proven that social environments are strongly associated with learners’ willingness to use an L2 for communication (Li et al., 2021; Peng, 2025). Within this line of inquiry, perceived social support is defined as learners’ subjective assessment of the affective, cognitive, and instrumental support they obtain from key sources such as teachers, peers, and family members (Zimet et al., 1988).

Perceived social support has consistently been linked to higher levels of motivation, engagement, and psychological well-being (Poudel et al., 2020; Wang et al., 2023). In EFL contexts, teacher and peer support have been shown to facilitate WTC by reducing anxiety and creating classroom environments that encourage communicative risk-taking (Ducker, 2021; Liu L. et al., 2024; Yang Y. et al., 2024). For example, Liu and Hong (2021) found that perceived social support positively predicted WTC among Chinese EFL learners, primarily through its role in lowering foreign language anxiety. Likewise, Zarrinabadi and Khodarahmi (2024) demonstrated that teacher support enhanced WTC by promoting both cognitive and affective engagement.

Although substantial evidence supports the relationship between perceived social support and WTC, most existing studies have primarily examined affective mediators, such as anxiety or enjoyment (Lee, 2022; Li C. et al., 2025; Wang and Hui, 2024). Comparatively, less attention has been paid to how perceived social support may enhance learners’ longer-term capacity to manage everyday academic challenges that could impede communication. This limitation highlights the need to consider adaptive psychological resources alongside affective mechanisms when explaining how social support influences communicative willingness.

### 2.3 WTC and academic buoyancy

Academic buoyancy captures students’ capability to handle routine academic challenges, including performance pressure, difficult tasks, and minor setbacks (Martin and Marsh, 2008). It is conceptually and empirically distinct from two related adaptive constructs discussed in SLA research: L2 resilience and L2 grit. Specifically, academic buoyancy primarily focuses on managing everyday and relatively minor academic adversities (Martin and Marsh, 2009; Putwain et al., 2020), which corresponds to the ongoing learning stresses commonly experienced in language classrooms. By contrast, L2 resilience generally concerns learners’ ability to overcome more severe or acute linguistic and academic challenges (Yun et al., 2018). Meanwhile, L2 grit captures a learner’s persistent effort and enduring enthusiasm for long-run language learning goals (Bensalem et al., 2025), highlighting commitment to distant objectives. Recent SLA research has further examined L2 grit alongside other learner characteristics, such as motivation, anxiety, and achievement, particularly in relation to growth-oriented beliefs and mindset interventions (Afsharmehr, 2026; Ansari, 2026). While these lines of research collectively reinforce the broader relevance of adaptive learner characteristics in SLA, they also underscore key differences in their primary focus (Martin and Marsh, 2009). Unlike recovery from major adversity or long-term persistence, academic buoyancy specifically targets immediate responses to daily academic hurdles as they arise. This specific focus renders it uniquely relevant to WTC, which is itself highly situational and context dependent (Lee, 2022; MacIntyre et al., 2019a).

Recent studies in EFL contexts have provided substantial empirical evidence linking academic buoyancy with a range of positive learning-related outcomes. Higher academic buoyancy has been associated with stronger motivational persistence, superior emotion regulation, and elevated classroom engagement (Xu and Zheng, 2025; Xu and Wang, 2022). Additionally, academic buoyancy correlates positively with self-efficacy (Yang S. et al., 2024) and classroom engagement (Liu H. et al., 2024), while exhibiting a negative association with academic burnout (Li X. et al., 2025). These findings suggest that learners who are better equipped to manage routine academic difficulties are more likely to maintain adaptive psychological and behavioral engagement. Emerging evidence further indicates that academic buoyancy may operate as a mediating mechanism through which contextual support promotes positive learning behaviors (Yang and Liang, 2025).

Although direct empirical research connecting academic buoyancy specifically with L2 WTC remains scarce, related evidence concerning other adaptive psychological resources provides a solid foundation for positing such a relationship. For instance, Amini et al. (2025) revealed that personal adaptive resources (resilience and perceived competence) mediated the links between social comparison orientations and L2 WTC among undergraduate EFL learners. While their findings focused on resilience rather than buoyancy, they provide strong theoretical support for the notion that learner-level adaptive resources explain how contextual perceptions relate to communicative willingness. This offers a compelling rationale for examining academic buoyancy as a potential mediator between perceived social support and L2 WTC in this study.

However, few prior studies have simultaneously investigated perceived social support, academic buoyancy, and WTC within a single integrated framework. Most previous research has either treated social support and academic buoyancy as independent predictors (Ibrahim and Basim, 2024) or focused primarily on affective mediators linking perceived social support to WTC (Yang Y. et al., 2024). Consequently, little empirical work has specifically investigated academic buoyancy as a psychological mechanism through which social support may strengthen EFL learners’ WTC.

This study attempts to explore the possible connection among these constructs. Given the multidimensional nature of perceived social support, the present study examines teacher, parent, and peer support separately to investigate their differentiated effects on academic buoyancy and WTC in the EFL context of China.

A structural model of academic buoyancy, WTC, and support from teachers, parents, and peers was proposed based on the aforementioned theoretical reasoning. Figure 1 shows a graphic representation of the model along with its paths. We proposed the following hypotheses:

**Figure 1:**
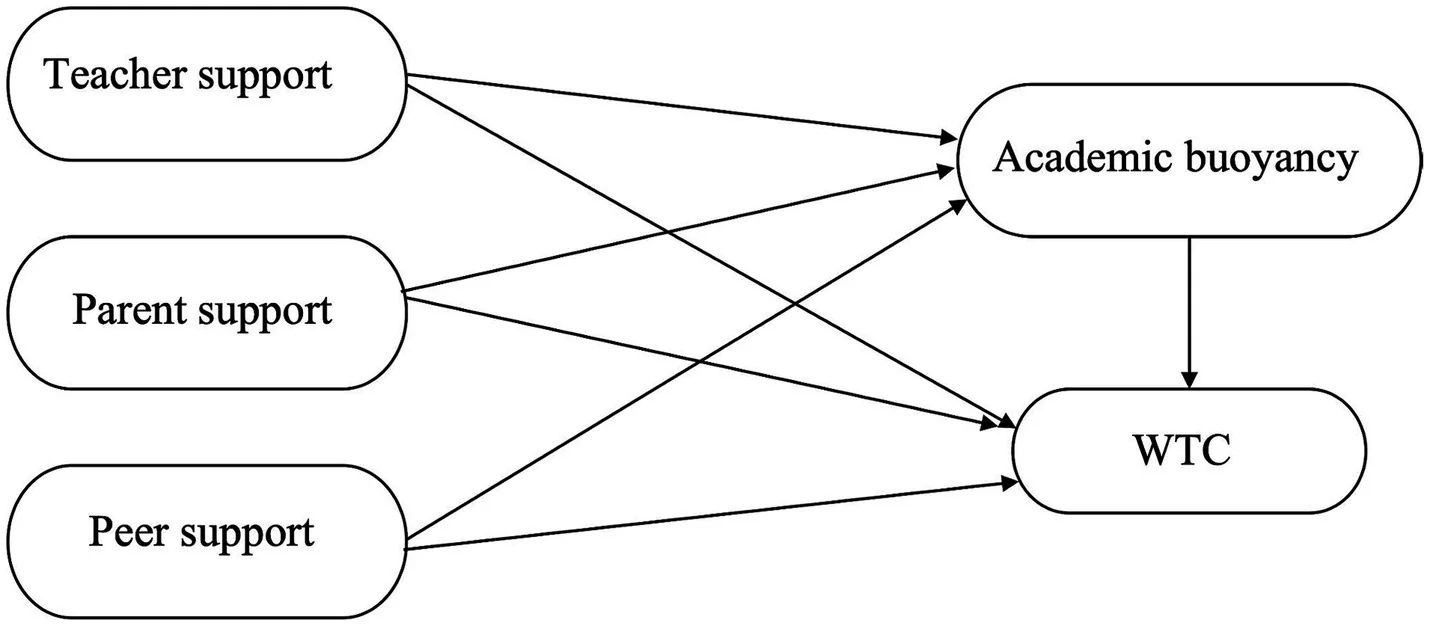
Hypothesized structural model.

*Hypothesis 1*: Teacher support positively predicts WTC.

*Hypothesis 2*: Parent support positively predicts WTC.

*Hypothesis 3*: Peer support positively predicts WTC.

*Hypothesis 4*: Teacher support positively predicts academic buoyancy.

*Hypothesis 5*: Parent support positively predicts academic buoyancy.

*Hypothesis 6*: Peer support positively predicts academic buoyancy.

*Hypothesis 7*: Academic buoyancy positively predicts WTC.

*Hypothesis 8*: Academic buoyancy mediates the relationship between teacher support and WTC.

*Hypothesis 9*: Academic buoyancy mediates the relationship between parent support and WTC.

*Hypothesis 10*: Academic buoyancy mediates the relationship between peer support and WTC.

## 3 Methodology

### 3.1 Participants and the setting

Using convenience sampling, we recruited first-year non-English-major undergraduates from a public university located in eastern China. A total of 374 questionnaires were collected. Five cases were removed because their response times were excessively short, leaving 369 valid cases and a valid response rate of 98.66%. All questionnaire items were mandatory, so the final dataset contained no missing values. The final sample of 369 participants provided an adequate basis for the planned analyses (Kline, 2023). The sample comprised exclusively freshmen enrolled in non-English degree programs, representing students in the initial stage of university study (Owusu-Agyeman and Mugume, 2023; Terenzini et al., 1994).

The final sample comprised 234 female participants (63.4%) and 135 male respondents (36.6%). Most individuals were 18 years old (59.1%) or 19 years old (30.6%). Regarding academic backgrounds, 48.5% of participants were from science-related disciplines, 40.1% from arts programs, and 11.4% from other fields including fine arts and physical education. On average, respondents had roughly 10 years of formal English learning experience. In addition, 99.2% of participants had no earlier experience of studying or living in English-speaking regions, indicating that the sample was predominantly situated in an EFL learning context.

### 3.2 Instruments

Three widely used self-report scales were adopted to measure the core constructs: WTC in English, perceived social support from teachers, parents, and peers, and academic buoyancy. All questionnaire items adopted a 5-point Likert scoring standard, ranging from 1 (strongly disagree) to 5 (strongly agree). To help participants fully understand the items, the questionnaires were presented in Chinese. The WTC and academic buoyancy scales were adapted from English into Chinese by the research team following a forward- and back-translation procedure, with minor discrepancies resolved to ensure semantic equivalence. The MSPSS was administered using an authorized Chinese version (Yang X. et al., 2024); therefore, no additional translation was performed for this scale.

#### 3.2.1 WTC scale

WTC in English was assessed using a 10-item scale developed by Peng and Woodrow (2010), designed to evaluate the readiness of learners to participate in various classroom-based oral activities, such as role plays and asking questions. This scale has been applied in EFL research and is considered a reliable indicator of situational WTC. A sample item is “I am willing to give a short self-introduction without notes in English to the class.” In this study, the scale had a Cronbach’s alpha of 0.915.

#### 3.2.2 The perceived social support scale

Perceived social support was measured with the authorized Chinese MSPSS (Yang X. et al., 2024). The original MSPSS contains family, friends, and significant-other subscales. Because the significant-other subscale does not specifically assess teacher support, the present authors adapted this subscale into a teacher-support subscale for the Chinese EFL context. This adaptation was based on the rationale that teachers are salient significant others for university EFL learners. Permission to use and adapt the scale was obtained from Yang X. et al. (2024). The adapted scale comprised three 4-item subscales: teacher support (α = 0.887), parent support (α = 0.900), and peer support (α = 0.864). A separate CFA of the 12 MSPSS items supported the three-factor structure in the present sample (*χ*^2^ = 127.449, df = 51, CFI = 0.971, TLI = 0.962, RMSEA = 0.064, SRMR = 0.022). Sample items include “My teacher is available when I need help,” “My parents are willing to help me when I have difficulties,” and “My friends are willing to share my joys and sorrows.” Full items and detailed CFA results are provided in Supplementary Tables S1, S2.

#### 3.2.3 The academic buoyancy scale

A 4-item scale from Martin and Marsh (2008) was adopted to measure academic buoyancy. The scale evaluates students’ capability to handle routine academic difficulties and minor frustrations. The scale has been widely adopted in EFL and higher education research and has shown satisfactory reliability and validity. A sample item is “I’m good at dealing with setbacks at school.” The internal consistency of this scale was reported to be 0.880.

### 3.3 Data collection

Data collection was conducted through online questionnaires distributed during regular class hours using Wenjuanxing, a widely used online survey application in China. The questionnaire combined the three scales described above. Informed consent was obtained electronically. The first page of the online questionnaire presented the study information sheet, including the study’s purpose, voluntary nature, confidentiality, anonymity, and the right to withdraw at any time without penalty. Participants could proceed to the questionnaire items only after clicking the “I agree/continue” button to indicate their consent. No questionnaire items were accessible to participants who did not provide consent.

### 3.4 Data analysis

SPSS 26.0 and AMOS 26.0 were utilized for data analysis and SEM, respectively. First, the dataset was screened for invalid responses, missing data, and distributional characteristics. Because all questionnaire items were mandatory, no missing values were present in the final dataset. Skewness and kurtosis were examined at both the variable and item levels to assess the suitability of the data for maximum-likelihood estimation. Second, internal consistency was assessed using Cronbach’s alpha. Confirmatory factor analysis (CFA) was then conducted in AMOS 26.0 to evaluate the measurement model. Model fit was evaluated using multiple indices rather than a single criterion. As commonly used guidelines, *χ*^2^/df values below 3.00, CFI and TLI values above 0.90, RMSEA and SRMR values below 0.08, and PCLOSE values above 0.05 were considered indicative of acceptable fit (Hu and Bentler, 1999). These values were treated as guidelines rather than rigid decision rules, and model fit was evaluated collectively in relation to model complexity and theoretical plausibility. Convergent validity was assessed using standardized factor loadings, composite reliability (CR), and average variance extracted (AVE) (Hair et al., 2010), while discriminant validity was examined using the Fornell–Larcker criterion and competing CFA models. Third, common method bias (CMB) was assessed using three complementary procedures. Harman’s single-factor test was conducted as an initial diagnostic, and the hypothesized five-factor measurement model was compared with models incorporating fewer factors. Finally, latent SEM was applied to test the proposed mediation model. In this study, teacher support, parent support, and peer support were operationalized as three distinct exogenous latent variables. Academic buoyancy was set as the mediator, and WTC as the dependent variable. Standardized path coefficients, standard errors, critical ratios, *p*-values, and explained variance (*R*^2^) were examined for the structural paths. Indirect effects were tested using 5,000 bootstrap resamples with 95% bias-corrected confidence intervals (Hayes, 2013). The hypothesized partial mediation model was subsequently compared with theoretically motivated alternative structural models, including a full mediation model specification, to examine the relative empirical compatibility of the proposed structural relationships.

## 4 Results

### 4.1 Preliminary analyses

Of the 374 questionnaires collected, five cases were excluded because of excessively short response times, resulting in 369 valid cases. Because the questionnaire items were mandatory, no missing values were present in the final dataset. Descriptive statistics, reliability coefficients, and distributional characteristics were examined before the measurement and structural models were tested. Cronbach’s alpha coefficients ranged from 0.864 to 0.915 across the study constructs, indicating satisfactory internal consistency. The teacher, parent, and peer support yielded coefficients of 0.887, 0.900, and 0.864, respectively. The Academic Buoyancy scale showed an alpha of 0.880, and the WTC scale yielded an alpha of 0.915. Normality was also examined. At the variable level, skewness ranged from −0.329 to 0.012 and kurtosis from −0.166 to 1.382 (Table 1). At the item level, skewness ranged from −0.346 to 0.230 and kurtosis from −0.479 to 0.899 (Table 2). These values indicated no substantial departures from univariate normality and supported the use of maximum-likelihood estimation in the subsequent CFA and SEM analyses.

**Table 1:** Variable-level skewness, kurtosis, means, standard deviations, and correlations among the study variables.

Variable	Descriptive statistics				Pearson correlations				
	Skewness	Kurtosis	M	SD	1	2	3	4	5
1. Teacher support	0.012	1.382	3.292	0.657	1				
2. Parent support	−0.093	−0.166	3.263	0.795	0.395**	1			
3. Peer support	−0.191	0.582	3.475	0.640	0.487**	0.288**	1		
4. Academic buoyancy	−0.264	1.137	3.310	0.705	0.505**	0.360**	0.424**	1	
5. WTC	−0.329	1.296	3.266	0.589	0.567**	0.457**	0.505**	0.482**	1

M, mean; SD, standard deviation; WTC, willingness to communicate in English. Correlations are Pearson’s *r* values based on composite observed scores. **p < 0.01 (two-tailed).

**Table 2:** Item-level skewness and kurtosis for all questionnaire items.

Item	Skewness	Kurtosis
WTC1	0.004	0.295
WTC2	0.024	−0.043
WTC3	−0.132	−0.078
WTC4	−0.142	0.414
WTC5	−0.069	−0.106
WTC6	−0.062	0.241
WTC7	−0.251	0.053
WTC8	−0.153	−0.325
WTC9	−0.080	−0.088
WTC10	−0.116	0.105
Teacher support 1	−0.083	0.264
Teacher support 2	−0.031	0.364
Teacher support 5	0.230	0.595
Teacher support 10	−0.167	0.899
Parent support 3	0.061	−0.440
Parent support 4	−0.261	−0.401
Parent support 8	0.055	−0.479
Parent support 11	0.006	0.108
Peer support 6	0.106	0.027
Peer support 7	−0.058	−0.019
Peer support 9	−0.204	−0.150
Peer support 12	−0.346	0.558
Academic buoyancy 1	−0.138	0.296
Academic buoyancy 2	−0.171	0.602
Academic buoyancy 3	−0.131	0.386
Academic buoyancy 4	−0.100	0.443

WTC, willingness to communicate in English. All item-level skewness and kurtosis values fell within the acceptable range for maximum-likelihood estimation.

### 4.2 Measurement model and construct validity

A five-factor CFA model was estimated in AMOS 26.0, with WTC, teacher support, parent support, peer support, and academic buoyancy specified as distinct latent constructs. The results demonstrated an acceptable overall fit to the data: *χ*^2^ = 507.587, *df* = 289, *p* < 0.001, *χ*^2^/df = 1.756, CFI = 0.961, TLI = 0.957, RMSEA = 0.045, SRMR = 0.037, AIC = 631.587, BIC = 874.057 (Table 3). NFI was marginally below 0.90, while all other core fit indices met conventional thresholds, supporting acceptable model fit. All standardized factor loadings reached statistical significance at the level of *p < 0.001* and ranged from 0.732 to 0.893. The composite reliability (CR) values ranged from 0.865 to 0.915, while average variance extracted (AVE) values varied from 0.518 to 0.692 (Table 4). These results provided evidence of adequate convergent validity across the five latent constructs.

**Table 3:** Goodness-of-fit indices for the measurement model and structural model.

Fit index	Measurement model (CFA)	Structural model (SEM)	Common reference value
$\chi^{2}$	507.587	507.587	–
df	289	289	–
*χ*^2^/df	1.756	1.756	$1<\chi^{2}/\mathrm{df}<3$
*p*	0.000	0.000	–
GFI	0.907	0.907	>0.90
NFI	0.887	0.887	>0.90
IFI	0.962	0.962	>0.90
CFI	0.961	0.961	>0.90
TLI	0.957	0.957	>0.90
RMSEA	0.045	0.045	<0.08
PCLOSE	0.880	0.880	>0.05
SRMR	0.037	0.037	<0.08
AIC	631.587	631.587	–
BIC	874.057	874.057	–

The structural model is a saturated structural model with the same number of free parameters as the CFA model; therefore, global fit indices are identical. NFI = 0.887 was slightly below the conventional 0.90 cutoff; however, the remaining fit indices indicated acceptable model fit. The marginally lower NFI did not materially compromise the overall adequacy of the model.

**Table 4:** Convergent validity, discriminant validity and correlations among constructs.

Construct	CR	AVE	1	2	3	4	5
WTC	0.915	0.518	**0.720**				
Teacher support	0.888	0.664	0.631	**0.815**			
Parent support	0.900	0.692	0.509	0.444	**0.832**		
Peer support	0.865	0.616	0.569	0.554	0.328	**0.785**	
Academic buoyancy	0.880	0.648	0.539	0.571	0.408	0.488	**0.805**

Bold values on the diagonal are the square root of the AVE for each construct; off-diagonal values are latent variable correlations from the five-factor CFA model. CR = composite reliability; AVE = average variance extracted; WTC = willingness to communicate in English.

The Fornell–Larcker approach was adopted to examine discriminant validity. As presented in Table 4, the square root of the AVE for each construct exceeded its correlations with the other latent constructs, supporting the empirical distinctiveness of the five constructs. Competing CFA models provided additional evidence for construct distinctiveness (Table 5). The hypothesized five-factor model showed substantially better fit than models combining two or more constructs. For example, the one-factor model showed poor fit, *χ*^2^/df = 8.271, CFI = 0.616, TLI = 0.582, RMSEA = 0.141, and SRMR = 0.105. The progressively poorer fit of the four-, three-, two-, and one-factor models was consistent with the specification of WTC, the three sources of social support, and academic buoyancy as distinguishable constructs.

**Table 5:** Competing CFA models for construct distinctiveness and common-method bias assessment.

Model	Factor structure	*χ*^2^/df	CFI	TLI	IFI	GFI	RMSEA	SRMR
Five-factor Model	WTC, Teacher Support, Parent Support, Peer Support, Academic Buoyancy	1.756	0.961	0.957	0.962	0.907	0.045	0.037
Four-factor Model	WTC + Teacher Support, Parent Support, Peer Support, Academic Buoyancy	3.343	0.879	0.865	0.865	0.797	0.080	0.061
Three-factor Model	WTC + Teacher Support + Parent Support, Peer Support, Academic Buoyancy	5.483	0.765	0.742	0.767	0.696	0.110	0.084
Two-factor Model	WTC + Teacher Support + Parent Support+ Peer Support, Academic Buoyancy	6.810	0.694	0.666	0.695	0.642	0.126	0.095
One-factor Model	WTC + Teacher Support + Parent Support+ Peer Support+ Academic Buoyancy	8.271	0.616	0.582	0.617	0.590	0.141	0.105
CMB Factor Model	–	1.700	0.967	0.960	0.968	0.916	0.044	0.036

CMB, common-method bias; WTC, willingness to communicate in English. The five-factor model represents the hypothesized measurement model. The CMB factor model includes an unmeasured latent common-method factor.

### 4.3 Assessment of common method bias

To evaluate potential common method variance, two complementary approaches were applied. First, Harman’s single-factor test indicated that the single factor accounted for 41.55% of the total variance, which was below the commonly used 50% reference point. This result did not suggest that a single common factor accounted for the majority of the observed variance. Then, an unmeasured common latent method factor was incorporated into the measurement model. The resulting model showed *χ*^2^/df = 1.700, CFI = 0.967, TLI = 0.960, RMSEA = 0.044, and SRMR = 0.036, compared with *χ*^2^/df = 1.756, CFI = 0.961, TLI = 0.957, RMSEA = 0.045, and SRMR = 0.037 for the five-factor model (Table 5). Although the common-method-factor model showed a modest improvement in some fit indices, the improvement was limited. Thus, the analyses did not provide evidence that common method bias substantially altered the measurement model, although its influence cannot be completely ruled out.

### 4.4 Correlation analysis

Correlations among the observed composite scores of teacher support, parent support, peer support, academic buoyancy and WTC were examined using Pearson’s correlation test. All three sources of support were positively correlated with academic buoyancy (Table 1). Specifically, teacher support showed a moderately positive correlation with academic buoyancy (*r* = 0.505, *p* < 0.01), followed by peer support (*r* = 0.424, *p* < 0.01) and parent support (*r* = 0.360, *p* < 0.01).

Similarly, teacher support showed the strongest observed-score correlation with WTC (*r* = 0.567, *p* < 0.01), followed by peer support (*r* = 0.505, *p* < 0.01) and parent support (*r* = 0.457, *p* < 0.01). Academic buoyancy was also positively correlated with WTC (r = 0.482, *p* < 0.01). These bivariate associations were consistent with the expected direction of relationships and provided a basis for proceeding to the multivariate structural analysis.

### 4.5 Structural model testing

The hypothesized structural relationships were tested using a structural equation modeling approach. We modeled teacher, parent, and peer support as correlated exogenous variables, with academic buoyancy positioned in an intermediary role and WTC designated as the final endogenous variable. Direct structural linkages were estimated from all three support domains toward both academic buoyancy and WTC, complemented by a direct trajectory from academic buoyancy to WTC.

The structural model produced the same global fit statistics as the five-factor CFA model: (*χ*^2^ = 507.587, df = 289, *χ*^2^/df = 1.756, CFI = 0.961, TLI = 0.957, RMSEA = 0.045, PCLOSE = 0.880, SRMR = 0.037) (Table 3). The identical fit statistics were expected because, with three correlated exogenous support constructs, one mediator, and one outcome, the specified recursive structural relations did not impose additional overidentifying restrictions on the covariance structure relative to the measurement model. Consequently, the global fit indices assess the reproduced covariance structure but do not by themselves establish the directional ordering of the structural paths.

The results of the model path hypothesis test (Figure 2) and Table 6 showed that teacher support was positively associated with both academic buoyancy (*β* = 0.370, SE = 0.067, CR = 5.406, *p* < 0.001) and WTC (β = 0.304, SE = 0.057, CR = 4.737, *p* < 0.001). Parent support was positively associated with academic buoyancy (*β* = 0.169, SE = 0.047, CR = 3.017, *p* = 0.003) and WTC (*β* = 0.231, SE = 0.039, CR = 4.536, *p* < 0.001). Similarly, peer support was also positively associated with academic buoyancy (*β* = 0.228, SE = 0.068, CR = 3.591, *p* < 0.001) and WTC (*β* = 0.253, SE = 0.058, CR = 4.349, *p* < 0.001). Furthermore, academic buoyancy was a significant positive predictor of WTC (*β* = 0.148, SE = 0.053, CR = 2.532, *p* = 0.011). The model explained 39.1% of the variance in academic buoyancy (*R*^2^ = 0.391) and 53.3% of the variance in WTC (*R*^2^ = 0.533). Thus, all hypothesized direct paths reached statistical significance in the specified model.

**Figure 2:**
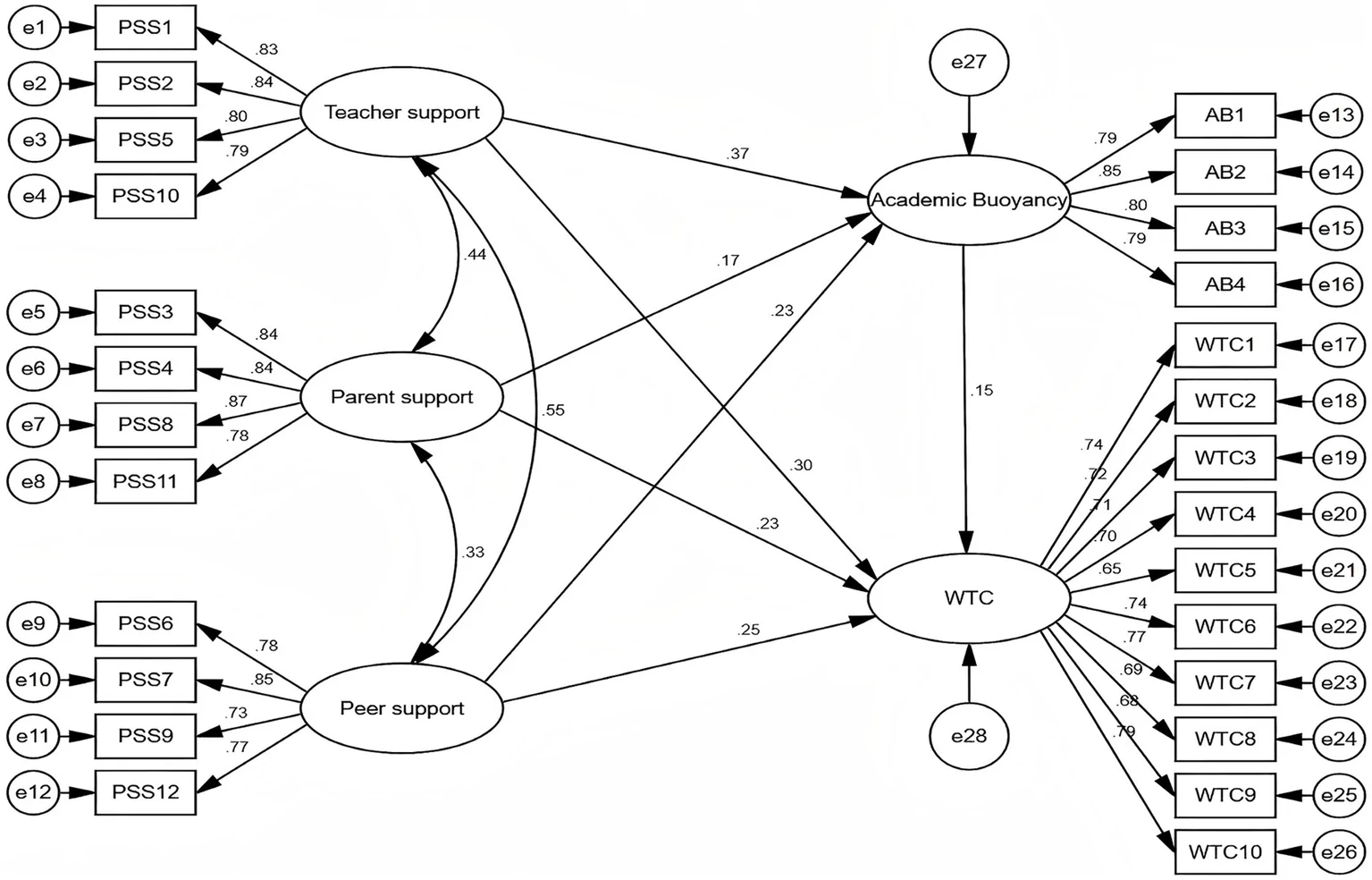
Standardized structural equation model.

**Table 6:** Standardized path coefficients for the structural model.

Direct path	*β*	SE	CR	*p*	*R* ^2^
Teacher support → Academic buoyancy	0.370	0.067	5.406	< 0.001	0.391
Parent support → Academic buoyancy	0.169	0.047	3.017	0.003	.
Peer support → Academic buoyancy	0.228	0.068	3.591	< 0.001	.
Teacher support → WTC	0.304	0.057	4.737	< 0.001	0.533
Parent support → WTC	0.231	0.039	4.536	< 0.001	.
Peer support → WTC	0.253	0.058	4.349	< 0.001	.
Academic buoyancy → WTC	0.148	0.053	2.532	0.011	.

The *R*^2^ value of 0.391 refers to academic buoyancy as the dependent variable, and the *R*^2^ value of 0.533 refers to WTC as the dependent variable.

### 4.6 Mediation analysis and alternative model comparison

To assess the indirect effects of teacher, parent, and peer support on WTC through academic buoyancy, we performed a bootstrapping procedure with 5,000 resamples. Bias-corrected 95% confidence intervals were examined to determine whether the indirect effects differed significantly from zero. As shown in Table 7, all three indirect effects were statistically significant.

**Table 7:** Bootstrap estimates of the total, direct, and indirect effects.

Path	Effect type	*β*	S.E.	95% CI		*p*	% of the total effect
				Lower	Upper		
Teacher support → Academic Buoyancy → WTC	Total Effect	0.359	0.084	0.191	0.517	0.000	-
	Direct Effect	0.304	0.089	0.126	0.476	0.001	84.68%
	Indirect Effect	0.055	0.028	0.010	0.124	0.016	15.32%
Parent support → Academic Buoyancy → WTC	Total Effect	0.256	0.062	0.134	0.377	0.000	-
	Direct Effect	0.231	0.060	0.114	0.356	0.000	90.23%
	Indirect Effect	0.025	0.017	0.002	0.071	0.024	9.77%
Peer support → Academic Buoyancy → WTC	Total Effect	0.287	0.073	0.147	0.433	0.000	-
	Direct Effect	0.253	0.078	0.102	0.408	0.001	88.11%
	Indirect Effect	0.034	0.022	0.004	0.098	0.016	11.89%

Bootstrap resamples = 5,000; WTC, willingness to communicate in English; CI, bias-corrected confidence interval.

For teacher support, the total effect on WTC was *β* = 0.359 (*p* < 0.001), the direct effect was *β* = 0.304 (*p* < 0.001), and the indirect effect through academic buoyancy was *β* = 0.055. The indirect effect accounted for 15.32% of the total effect. For peer support, the total effect was *β* = 0.287 (*p* < 0.001), the direct effect was *β = 0.253,* and the indirect effect was *β* = 0.034, accounting for 11.89% of the total effect. For parent support, the total effect was *β* = 0.256 (*p* < 0.001), with a direct effect of *β* = 0.231 (*p* < 0.001), and an indirect effect of *β* = 0.025, accounting for 9.77% of the total effect. Given that the 95% confidence intervals across all three indirect effects did not include zero and the direct effects remained significant, the results were consistent with partial statistical mediation.

To examine whether the hypothesized structural specification provided a better empirical account than a theoretically plausible alternative, we compared the hypothesized partial mediation model with a full mediation model, in which the direct paths from the three sources of social support to WTC were constrained to zero. Model fit was evaluated using *χ*^2^, df, CFI, TLI, RMSEA, SRMR, AIC, and BIC, with detailed results reported in Table 8. The full mediation model yielded a significantly worse fit than the partial mediation model, Δ*χ*^2^ (3) = 107.503, *p* < 0.001. Furthermore, the partial mediation model demonstrated superior goodness-of-fit indices (CFI = 0.961 vs. 0.943; TLI = 0.957 vs. 0.936; RMSEA = 0.045 vs. 0.055; SRMR = 0.037 vs. 0.061) and lower information criteria (AIC = 631.587 vs. 733.090; BIC = 874.057 vs. 963.827). Although the full mediation model showed a slightly higher *R*^2^ for academic buoyancy (0.444 vs. 0.391), the partial mediation model accounted for substantially more variance in the primary outcome, WTC (*R*^2^ = 0.533 vs. 0.350).

**Table 8:** Comparison of the hypothesized partial mediation model and the full mediation model.

Fit index	Partial mediation model (hypothesized)	Full mediation model
$\chi^{2}$	507.587	615.090
df	289	292
*χ*^2^/df	1.756	2.106
Δ*χ*^2^ (Δdf)	–	107.503(3)
*p*	0.000	0.000
GFI	0.907	0.891
NFI	0.887	0.869
IFI	0.962	0.897
CFI	0.961	0.943
TLI	0.957	0.936
RMSEA	0.045	0.055
SRMR	0.037	0.061
AIC	631.587	733.090
BIC	874.057	963.827
*R*^2^ (Academic buoyancy)	0.391	0.444
*R*^2^ (WTC)	0.533	0.350

*R*^2^ values are model-specific, equation-level estimates for each endogenous variable and should be interpreted together with the overall model-fit indices.

Importantly, *R*^2^ is model- and outcome-specific and should not be interpreted as an index of overall model adequacy. The two models used different structural specifications, and the *R*^2^ values therefore represent model-specific estimates. The higher *R*^2^ for academic buoyancy in the full-mediation model (0.444 vs. 0.391) does not indicate better overall model fit or greater explanatory power for the focal outcome. For WTC, the full-mediation model explained 35.0% of the variance, compared with 53.3% in the partial-mediation model, a difference of 18.3 percentage points. This lower explanatory power is consistent with the full mediation model constraining the three direct support-to-WTC paths to zero, whereas these paths were freely estimated in the partial mediation model. Thus, the higher model-specific *R*^2^ for academic buoyancy does not contradict the full-mediation model’s lower explanatory power for WTC or its poorer overall fit. Based on the significant chi-square difference and the more favorable fit indices and information criteria, the partial mediation model was retained. Given the cross-sectional design, this model comparison is interpreted as evidence of relative empirical fit to the observed data rather than definitive evidence of causal or temporal ordering.

## 5 Discussion

The study examined how academic buoyancy was related to the associations of teacher, parent, and peer support with WTC among Chinese EFL undergraduates. Using SEM with bootstrapping, the findings provided empirical support for all hypothesized direct and indirect relationships across the three sources of social support. Disaggregated data showed that each type of social support was differentially associated with academic buoyancy and WTC. This outcome helps build a more context-aware interpretation of learners’ WTC.

Overall, the findings suggest that external social resources interact with learners’ internal adaptive traits to jointly predict their WTC. Integrating academic buoyancy into established WTC frameworks enables a more holistic understanding of communicative readiness. Further evidence points to the notion that adaptive coping works in tandem with affective and cognitive factors to foster learners’ willingness to engage in L2 interaction. As such, academic buoyancy appears to act as a vital psychological link that may facilitate the translation of perceived social support into consistent communicative participation. The results also revealed varying strength across different support providers, with teacher support exhibiting the most substantial association, followed by peer and parent support. By clarifying the dynamic relationship between contextual support and academic buoyancy in L2 communication, this research further enriches existing theoretical models in this field.

### 5.1 Three sources of perceived social support as direct predictors of WTC (H1–H3 supported)

As predicted in Hypotheses 1–3, teacher, parent, and peer support were positively associated with WTC. This finding accords with the ecological view of WTC, which regards it not as a static disposition but as a contextually sensitive variable and suggests that social surroundings are related to individual communicative tendencies (Jamalvandi et al., 2020; Khajavy et al., 2018; Liu L. et al., 2024; Peng, 2012; Zhang et al., 2024). Empirically, this finding also resonates with previous SLA research, where supportive classroom environments tend to foster learners’ communicative engagement and WTC (Li C. et al., 2025; Liu H. et al., 2024; Zarrinabadi and Khodarahmi, 2024). Notably, the direct association of perceived social support with WTC still held after academic buoyancy was included as a mediating variable. This may imply that social support acts as a fundamental contextual resource, one that could mitigate psychological barriers in L2 interaction (Tu, 2021). Such function appears particularly relevant for EFL learners in China, a setting widely noted for prevailing face concerns and fear of negative evaluation (Li et al., 2023).

The relatively stronger role of teacher support may be partly understood in relation to the institutional position of teachers in Chinese tertiary EFL classrooms. In local classrooms, teachers generally take charge of instructional activities and largely influence the overall learning atmosphere (Cortazzi and Jin, 1996; Wen and Clément, 2003). Although slightly weaker than teacher support, peer support remains meaningful, likely through mechanisms such as collaborative learning, peer modeling, and the alleviation of interpersonal anxiety during pair or group activities (Ducker, 2021; Liu L. et al., 2024; Zarrinabadi and Khodarahmi, 2024). The descriptively larger coefficient for peer support than parent support may suggest a stronger association with communicative readiness, although the difference between these coefficients was not formally tested.

These findings from previous studies have predominantly emphasized teacher support (e.g., Hejazi et al. (2023); Liu L. et al. (2024); Ma and Xia (2026); MacIntyre et al. (2001); Peng (2020)), demonstrating that not all sources of social support exert the same influence. By disaggregating teacher, peer, and parent support, teacher support showed the strongest associations descriptively with WTC among Chinese EFL learners, reflecting the hierarchical, exam-oriented nature of the educational environment (Wen and Clément, 2003). While parent support yielded a relatively smaller direct effect, it remains a valuable source of encouragement for language learners. Instead of exerting influence via in-class interactions, parent support may function indirectly by shaping learners’ long-term motivation and emotional states, thereby affecting their WTC (MacIntyre et al., 2001).

### 5.2 Three sources of perceived social support as predictors of academic buoyancy (H4–H6 supported)

Hypotheses 4–6 were supported, with teacher, parent, and peer support each positively associated with academic buoyancy. The relatively stronger association observed for teacher support suggests that learners’ perceptions of support within the immediate academic environment may be particularly relevant to their perceived capacity to deal with routine academic difficulties. This interpretation aligns with the principles of the conservation of resources theory (Hobfoll, 1989), which posits that external social resources can be internalized to enhance learners’ psychological capacity to manage everyday academic challenges.

For first-year university students navigating a critical academic transition (Li et al., 2023), perceived support from teachers, peers, and parents serves as an essential external resource that fosters confidence in handling routine EFL learning challenges, including linguistic difficulties in speaking, performance pressure during classroom presentations, and minor setbacks in language assessments (Xu and Wang, 2022). These findings extend educational psychology research on the social antecedents of academic buoyancy to the SLA context (Li et al., 2024; Putwain et al., 2020) and support Yun et al.’s (2018) argument that academic buoyancy in the L2 classroom is context-sensitive rather than purely dispositional. Moreover, the results complement the framework proposed by Zhang et al. (2024) by introducing an adaptive capacity construct shaped by social contextual influences.

### 5.3 Academic buoyancy as a predictor of WTC (H7 supported)

In line with Hypothesis 7, academic buoyancy was positively associated with WTC. This result points to the contribution of adaptive coping capacity, which operates alongside more commonly studied affective variables including enjoyment and anxiety (Li C. et al., 2025; MacIntyre and Gregersen, 2022), as well as cognitive factors such as perceived communicative competence and growth mindset (Hejazi et al., 2023; Zhang et al., 2024). The observed pattern accords with a body of prior research that connects academic buoyancy to favorable learning outcomes in L2 contexts (Bai and Hu, 2025). It also suggests this adaptive capacity can serve as a functional mechanism to sustain learners’ WTC. This study advances extant literature by providing evidence of a direct predictive association between academic buoyancy and WTC. Learners with greater academic buoyancy can better manage negative emotions when facing communication difficulties, reframe difficulties as manageable setbacks, and continue participating orally despite temporary failures (Martin and Marsh, 2008; Yang and Liang, 2025). This finding also addresses the research appeals within SLA scholarship to shift focus away from short-term emotional states. Instead, scholars have urged greater attention to stable ability-based constructs that help learners maintain consistent engagement in L2 communication (Dewaele and MacIntyre, 2024; Souzandehfar and Ahmed Abdel-Al Ibrahim, 2023).

### 5.4 Partial mediating role of academic buoyancy (H8–H10 supported)

Hypotheses 8–10 were supported, as academic buoyancy partially mediated the relationship between all three sources of social support and WTC. The mediation findings are valuable because they suggest that the link between social support and communicative willingness may involve more than the immediate interpersonal environment. Learners’ adaptive responses to routine academic difficulties may represent one psychological pathway through which perceived social resources are aligned with WTC.

These results offer empirical support for extending MacIntyre et al.’s (1998, 2019b) multi-layered WTC framework, which categorizes antecedents into distal individual traits, intermediate psychological factors, and proximal environmental influences. Traditionally, the intermediate tier emphasized immediate affective and cognitive variables, such as perceived communicative competence and anxiety (Lee, 2022; MacIntyre and Gregersen, 2022). This study suggests a plausible extension of this framework by demonstrating that academic buoyancy, a relatively stable adaptive psychological capacity, may also function as a critical intermediate factor, mediating the relationship between proximal environmental resources and WTC.

Notably, while the direct relationships between social support and WTC constituted the primary share of the overall association, the indirect pathways through academic buoyancy also demonstrated statistical significance. The present data also hints that adaptive coping ability may act as a meaningful indirect mechanism connecting social support with learners’ WTC (Acosta-Manzano et al., 2024; Wang and Hui, 2024). In line with observations reported by Yang and Liang (2025), who noted that academic buoyancy mediates links between contextual resources and learning-related outcomes, this study extends this line of reasoning to the domain of communicative willingness.

When viewed alongside the cognitive and affective antecedent model of WTC developed by Zhang et al. (2024), the present findings underline the combined importance of social contextual conditions and personal adaptive resources. The current evidence lends further support to the widely shared view that WTC is associated with multiple interacting forces and is embedded in the social contexts where learning takes place (Dewaele, 2019; Peng, 2025).

Results from this study also suggest that academic buoyancy may be understood as a form of adaptive communicative agency (Yun et al., 2018). Beyond supporting emotional stability, this quality appears to help learners interpret communication-related challenges as routine academic obstacles rather than overwhelming threats (Bai and Hu, 2025). This kind of constructive interpretation may be especially important for sustained classroom participation in EFL contexts strongly shaped by sociocultural expectations, where public oral performance is often exposed to evaluative scrutiny from others. Perceived social support may help establish a secure psychological environment, while academic buoyancy provides the internal resources needed for learners to act confidently within that environment (Lin et al., 2025). Through this combined process, external supportive conditions can be gradually translated into consistent and active communicative engagement.

## 6 Pedagogical implications

The present study offers a few contextually responsive and theoretically informed pedagogical suggestions for supporting WTC among EFL learners. One central implication relates to the role of perceived social support in classroom dynamics. When viewed from a social-ecological lens, language classrooms can be understood not only as spaces for receiving linguistic input, but also as relational environments in which interpersonal connections shape students’ decisions to participate orally (Peng, 2012). For instructors, this means intentionally creating a classroom atmosphere characterized by emotional security and reduced evaluative stress. In culturally sensitive contexts such as China, where learners often express strong concerns over face maintenance, modest yet consistent classroom practices may help reduce unnecessary anxiety. These practices can include supportive responses to student contributions, genuine affirmation of participation regardless of linguistic accuracy, and the framing of errors as natural steps in language development. Over time, such actions may help learners feel safer and more prepared to initiate communication.

The mediating function of academic buoyancy also adds an important layer to instructional design. Learners often need not only external support but also internal psychological resources to manage the everyday challenges of speaking in an L2 (Yun et al., 2018). The broaden-and-build theory states that supportive social environments may help develop such adaptive qualities, which in turn enable learners to sustain engagement when faced with setbacks (Fredrickson, 2004). For practical teaching, this could mean shifting focus away from outcome-oriented evaluation toward process-oriented support. Useful strategies may involve designing communication tasks with gradually increasing difficulty, providing opportunities for repeated practice and reflective thinking, and modeling constructive responses to communication failures. When teachers help learners view struggle as a typical part of language learning and emphasize effort over perfection, students may be more likely to maintain long-term willingness to speak in class.

Differences observed among teacher, parent, and peer support also suggest the value of building a multi-layered support system. Within the physical classroom, structured peer activities, including collaborative tasks, interactive dialogues, and group problem-solving, may strengthen peer relationships while increasing opportunities for meaningful communication (Li Z. et al., 2025). Beyond classroom settings, institutions and teaching staff may consider engaging with families to foster a more nuanced recognition of socio-affective dynamics within the language learning process. Such efforts can help strengthen the broader support system surrounding individual learners.

These implications may also extend to online and blended learning environments, which have become increasingly common in higher education. Digital tools can either support or restrict interaction, largely based on how teachers design and implement learning activities (Wang et al., 2026). Teachers may therefore take deliberate steps to strengthen social presence and emotional connection in virtual spaces (Peng, 2025). Examples include structured online discussions, collaborative digital assignments, and timely personalized feedback. Supporting learners in overcoming common online challenges, such as reduced immediacy or increased self-consciousness, may encourage more active communication across different learning formats.

Overall, the promotion of L2 WTC may benefit from a holistic and integrated approach. Such an approach can combine positive interpersonal classroom dynamics, instructional practices that foster adaptive capacities, and task design adjusted to specific contextual needs. When educators address social and psychological factors alongside linguistic development, learners may gradually develop greater confidence and become more active communicators. In turn, these practices can help advance research on L2 communicative development by reinforcing socially situated and process-based understandings of language learning.

## 7 Limitations

Several limitations should be considered when interpreting the findings of this study. First, the cross-sectional design does not permit causal inferences or establish the temporal ordering of the relationships among perceived social support, academic buoyancy, and WTC. Although the SEM results provide evidence of statistically significant direct and indirect associations within the hypothesized model, they should not be interpreted as evidence of causal effects. Second, the study relied on self-reported questionnaire data collected from the same participants at a single time point. Although several procedures were used to assess potential common method bias, including Harman’s single-factor test and a common latent method factor model, the possibility of method-related influences cannot be completely excluded. Third, the participants were recruited from a single public university in eastern China using convenience sampling. The sample consisted exclusively of first-year non-English-major undergraduates, which may limit the transferability of the findings to students from other universities, educational levels, academic disciplines, or sociocultural contexts. Future research could address these limitations by adopting longitudinal or experimental designs to examine the temporal and causal relationships among the variables. Multi-site sampling and more diverse participant groups could also improve the generalizability of the findings. In addition, mixed-methods research could provide more detailed insight into how learners perceive and experience social support and academic buoyancy in different communicative contexts.

## 8 Conclusion

This study focused on a conceptual model designed to explore connections between perceived social support, derived from teachers, parents, and peers, and WTC among EFL learners. The SEM results showed that all three sources of social support were positively associated with both academic buoyancy and WTC. Academic buoyancy was also positively associated with WTC, and its indirect effects partially mediated the associations between each source of social support and WTC. The findings extend existing WTC research by incorporating academic buoyancy as an adaptive psychological construct within a socially embedded framework. They also provide a differentiated view of social support by examining teacher, parent, and peer support separately rather than treating social support as a single undifferentiated source. Overall, the findings suggest that learners’ communicative willingness is associated with both contextual social resources and adaptive psychological capacities. By positioning academic buoyancy alongside established affective and cognitive antecedents of WTC, this study provides an additional perspective for understanding the psychological and contextual factors related to L2 communicative engagement among EFL learners.

## Data Availability

The original contributions presented in the study are included in the article/Supplementary material, further inquiries can be directed to the corresponding author.
