# Supplementary material for: Perceived social support, academic buoyancy, and L2 willingness to communicate among Chinese EFL undergraduates: a structural equation modeling study
Source: Front Psychol. 2026 Oct 19;17:1920700. doi: 10.3389/fpsyg.2026.1920700 (PMC13724334; doi:10.3389/fpsyg.2026.1920700)
Supplement: Supplementary file 1 [file Table_1.DOCX]

Table S1 Adapted Chinese version of MSPSS

| **题号** | **题项** | **维度** |
| --- | --- | --- |
| 1 | 当我有困难的时候，老师会陪伴在我身边。 | 教师支持 |
| 2 | 我可以和老师分享我的喜怒哀乐。 | 教师支持 |
| 3 | 我的家人真心尽力帮助我。 | 父母支持 |
| 4 | 我从家人那里获得所需要的情感帮助与支持。 | 父母支持 |
| 5 | 老师能真正安慰我。 | 教师支持 |
| 6 | 我的朋友真心尽力帮助我。 | 同伴支持 |
| 7 | 遇到困难时，我可以依靠我的朋友。 | 同伴支持 |
| 8 | 我可以和家人谈论我的难题。 | 父母支持 |
| 9 | 我有可以一起分享喜怒哀乐的朋友。 | 同伴支持 |
| 10 | 在我的生活里，老师会在意我的感受。 | 教师支持 |
| 11 | 我的家人愿意帮我做决定。 | 父母支持 |
| 12 | 我可以和朋友谈论我的难题。 | 同伴支持 |

Source: Yang, X., Xue, M., Pauen, S., and He, H. (2024b). Psychometric properties of the Chinese version of multidimensional scale of perceived social support. *Psychology Research and Behavior Management* 17, 2233-2241. doi: 10.2147/PRBM.S463245.
