# Supplementary material for: Perceived social support, academic buoyancy, and L2 willingness to communicate among Chinese EFL undergraduates: a structural equation modeling study
Source: Front Psychol. 2026 Oct 19;17:1920700. doi: 10.3389/fpsyg.2026.1920700 (PMC13724334; doi:10.3389/fpsyg.2026.1920700)
Supplement: Supplementary file 2 [file Table_2.DOCX]

**Table S2_1. CFA model fitting index.**

| [Reference](D:/Program%20Files/Youdao/Dict/8.9.6.0/resultui/html/index.html#/javascript:;) [index](D:/Program%20Files/Youdao/Dict/8.9.6.0/resultui/html/index.html#/javascript:;) | Standard values | Statistic |
| --- | --- | --- |
| *X²* | - | 127.449 |
| *df* | - | 51 |
| *X²/df* | ＜3 | 2.499 |
| *P* | - | .000 |
| GFI | ＞.9 | .945 |
| NFI | ＞.9 | .916 |
| IFI | ＞.9 | .971 |
| CFI | ＞.9 | .971 |
| TLI | ＞.9 | .962 |
| RMSEA | ＜.08 | .064 |
| SRMR | ＜.08 | .022 |
| AIC | - | 181.449 |
| BIC | - | 287.040 |

**Table S2_2. Standardized factor loadings, reliability, convergent validity, and discriminant validity.**

| Construct | Item | Standardized Estimate | S.E. | t-value | *P* | CR | AVE |
| --- | --- | --- | --- | --- | --- | --- | --- |
| Teacher | PSS1 | .830 |  |  |  | .888 | .664 |
|  | PSS2 | .837 | .055 | 18.300 | *** |  |  |
|  | PSS5 | .801 | .050 | 17.297 | *** |  |  |
|  | PSS10 | .792 | .050 | 17.021 | *** |  |  |
| Parent | PSS3 | .842 |  |  |  | .900 | .692 |
|  | PSS4 | .838 | .053 | 19.068 | *** |  |  |
|  | PSS8 | .864 | .051 | 19.899 | *** |  |  |
|  | PSS11 | .782 | .049 | 17.293 | *** |  |  |
| Peer | PSS6 | .787 |  |  |  | .865 | .616 |
|  | PSS7 | .853 | .064 | 16.735 | *** |  |  |
|  | PSS9 | .728 | .066 | 14.172 | *** |  |  |
|  | PSS12 | .767 | .061 | 15.051 | *** |  |  |

**Table S3_3 Discriminant validity**

| Discriminant validity | Teacher | Parent | Peer |
| --- | --- | --- | --- |
| Teacher | **.815** |  |  |
| Parent | .444 | **.832** |  |
| Peer | .554 | .328 | **.785** |

*Note.* CR = composite reliability; AVE = average variance extracted. In the lower panel, bold values on the diagonal are the square root of AVE; off-diagonal values are latent variable correlations from the CFA model. ****p* < .001.
